# The effect of signaling in dependence on the extraneous cognitive load in learning environments

**DOI:** 10.1007/s10339-020-01002-5

**Published:** 2020-10-27

**Authors:** Maik Beege, Steve Nebel, Sascha Schneider, Günter Daniel Rey

**Affiliations:** Psychology of Learning with Digital Media, Faculty of Humanities, Chemnitz University of Technology, Straße der Nationen 12, 09111 Chemnitz, Germany

**Keywords:** Instructional texts, Signaling, Disfluency, Cognitive load, Learning

## Abstract

Text-based learning media are often used in primary, secondary and university education. Therefore, text designers can support the learner by highlighting the most relevant information by using visual cues. Despite this signaling effect’s broad empirical basis, the extent to which the effectiveness of educational signals is dependent on moderator variables, like the design and layout of the text has not been investigated to date. In the current experiment, 138 university students learned about the formation of tsunamis from an instructional text. The text was manipulated in terms of *signaling* (color cues vs. no color cues) and *induced learning-irrelevant extraneous cognitive load* (fluent text font vs. disfluent text font). The results revealed that learners who had received the signaled text outperformed those who received the non-signaled text in terms of transfer performance. These results are explained by cognitive load, which was reduced in the signaling condition. The text font had no influence on the learning outcomes. Extraneous load induction further led to higher metacognitive accuracy and invested effort, while cognitive load and frustration were also increased. Interaction effects only occurred in terms of testing time, ease of learning and navigation. Results indicate that signaling is beneficial for transfer performance, independent of the font design of text.

## Introduction

When considering complex instructional texts, it is difficult to distinguish between learning-relevant and learning-irrelevant information. Learners who do not receive instructional help can easily be overwhelmed when trying to identify core information which is necessary for understanding the learning content (e.g., Anderson and Armbruster 1984; Mayer 2005). The signaling principle is a prominent design recommendation for supporting learners. Relevant information should be cued, in order to guide the learner’s attention to the most important parts of the text (van Gog 2014). This instructional support is especially necessary when the learning environment has a confusing or detrimental design (Sweller 2010). How the extraneous cognitive load (processing learning-irrelevant information due to suboptimal instructional design) induced through an instructional text influences the effects of signaling is not yet known. Therefore, the current research aims to investigate the signaling effect in interdependence on the legibility of the text, considering the disfluency effect. The aim is to clarify whether signaling is especially beneficial in instructional texts which induce a high cognitive load and are perceived to be very difficult.

## Literature review

### The signaling effect

The signaling or cueing principle refers to the finding that learning from instructional materials is enhanced when relevant elements or the organization of the material, are highlighted (for reviews, see Schneider et al. 2018; Richter et al. 2016; van Gog 2014). According to van Gog (2014), it is possible to distinguish two signaling modes: signaling within texts and signaling within pictures (including diagrams and animations). The current investigation focused on textual signaling, which can be divided into five types: organizational signals (e.g., headings or summaries), colors (e.g., font colors), text–picture references (e.g., “see the picture”), intonation (e.g., in auditory texts) or a mixture of types (e.g., coloring and text–picture referencing) (van Gog 2014).

The signaling principle can be explained considering the cognitive load theory (CLT; e.g., Paas and Sweller 2014; Sweller 1988). The CLT postulates that the total cognitive capacity is limited. According to recent approaches (Kalyuga and Singh 2016; Kalyuga and Plass 2017), two categories of cognitive load, concerned with the acquisition, storage and retrieval of information, can be distinguished. The first major type is the intrinsic (productive) cognitive load, which is the unavoidable load necessary for accomplishing a specific goal (Kalyuga and Plass 2017). The load arises from the cognitive processing of learning-relevant information and depends on the learner’s prior knowledge (Sweller et al. 2011). Germane processes, or the germane load, which are relevant for schema construction are subsumed under the productive load construct. The second major type is the extraneous (unproductive) cognitive load, which is not directly related to the achievement of a specific learning goal (Kalyuga and Plass 2017). The load arises from cognitive processing of learning-irrelevant information due to suboptimal instructional design (Mayer and Moreno 2010). With respect to CLT, signaling supports the learner by helping him to recognize which information in new instructional material is learning-relevant and which might be irrelevant for the learning goal. Signaling directs the learner’s attention to the most important, learning-relevant material. Especially in long and homogeneous texts, the selection of relevant information must be coordinated by using attention-guiding features within the instructional texts, to prevent cognitive overload. Eye-tracking studies pointed out that visual cues can direct the eye movements of learners to the most important information and limit the amount of time spent fixating the less relevant areas (e.g., Jamet 2014). Therefore, the signaling principle is also referred to as the attention-guiding principle. In consequence, signaling reduces unproductive extraneous load and increases productive intrinsic load (e.g., Amadieu et al. 2011).

In accordance with the theories described, studies have shown that signaling can enhance retention (e.g., Boucheix et al. 2013), and transfer performance during problem-based tasks (e.g., Liu et al. 2013). In addition, signaling also affects motivational and affective states (Schneider et al. 2018). Studies have shown that signaling decreases the learners’ stress levels (e.g., Skuballa et al. 2012), increases their enjoyment of the learning material (e.g., Johnson et al. 2015), increases the material’s attractiveness (e.g., Huk et al. 2003) and enhances their intrinsic motivation (Lin 2011).

### Moderators of the signaling effect

Recent meta-analyses regarding the signaling effect have postulated moderator effects in terms of the signaling mode (text vs. graphic), types of signaling (color vs. labeling vs. pointing gestures, etc.), instructional domain (natural sciences vs. math vs. history, etc.), the level of prior knowledge (high vs. low) and the pacing (self-paced vs. system-paced) (Richter et al. 2016; Schneider et al. 2018). For example, Schneider and colleagues (2018) argued that learners with prior knowledge are better able to cope with the additional information provided by signaling than those with low prior knowledge, and thus, signaling is especially beneficial for high-prior-knowledge learners. Nevertheless, low-prior-knowledge learners benefit from the attention-guiding cues in learning environments as well.

However, so far, less attention has been paid to the influence of cognitive load induced through the general design of the learning environment, on the effects of signaling. The concept of *element interactivity* is the focal point here. An element is any information that must be processed (Sweller 1994). In material with low element interactivity, every element can be processed with no or minimal reference to other elements (for example: the acquisition of a language’s vocabulary). High-element-interactivity material includes elements that interact strongly with each other (for example: the acquisition of a language’s grammar); the elements must be processed simultaneously for the learning content to be fully understood. The higher the number of elements interacts with each other, the higher the cognitive load on the working memory (Tindall-Ford et al. 1997). According to Sweller (2010), “element interactivity is the major source of working memory load underlying extraneous as well as intrinsic cognitive load” (p. 125). Design features in learning environments can be viewed as additional elements, which interact with the actual content and inevitably have to be processed (Beckmann 2010). Whether these features can be viewed as intrinsic load or extraneous load depends on the learning goal (Schnotz and Kürschner 2007). If an instructional text is written in an illegible font which makes it hard to process the information, the font can be viewed as an extraneous load. However, if the learning scenario’s goal is to learn this particular font, the load can be viewed as intrinsic. In both scenarios, the font contributes to the element interactivity because it is an additional element, which must be processed in interaction with the actual content (Sweller et al. 2019). Based on this definition, the *element interactivity effect* postulates that instructional support through signaling should be especially beneficial in learning materials which induce a high extraneous cognitive load. This support is not necessary in low-element-interactivity materials since learners still have resources available for dealing with the material’s suboptimal design (Chen et al. 2015, 2017).

### Induction of extraneous load

For the current research, extraneous load is induced through the legibility or perceptual disfluency of the text. Perceptual disfluency can be induced in instructional text, for example by illegible fonts (e.g., Besken and Mulligan 2013; Carpenter et al. 2013; Katzir et al. 2013; Pieger et al. 2016). According to the CLT, harder-to-read texts induce extraneous cognitive load, since additional cognitive resources are needed to decipher the illegible way in which the information is presented. This was supported by a recent study by Seufert et al. (2017), which indicated that strongly disfluent texts led to a greatly reduced legibility and increased extraneous cognitive load. According to Sweller’s definition of element interactivity (2010), the disfluent font can be viewed as an extraneous source of element interactivity, since the font’s acquisition is not the goal of the learning task.

It is especially important that disfluency must be particularly strong if it is supposed to induce a high cognitive load (Seufert et al. 2017). A low or moderate degree of disfluency can have the exact opposite effect (e.g., French et al. 2013; Weltman and Eakin 2014). In this context, disfluency can be viewed as a desirable difficulty (Bjork 1994). Difficult learning conditions can foster learning processes because the challenge posed by a disfluent learning environment stimulates cognitive engagement and fosters deeper cognitive processing and more elaborate strategies (e.g., Alter et al. 2007; Bjork 1994). Thus, metacognitive activities should be taken into account and measured, in order to determine whether extraneous load induction could act as a trigger for beneficial activities. In this case, extraneous load induction would not generally contribute to the element interactivity defined by Sweller (2010); extraneous elements trigger activities that might compensate for the cognitive processing difficulties induced by the design of the learning environment. Three main concepts are measured when discussing metacognitive activities in learning contexts (Nelson and Narens 1990): Ease of learning judgments is made before learning and affect the allocation of study time (Son and Kornell 2008). Ease of learning judgments is particularly affected by perceptual fluency, since no information about the complexity of the learning content is available at the time the judgment is made. Judgments of learning are made after learning from the text and predict future memory performance (Dunlosky and Metcalfe 2009). Finally, retrospective confidence assesses the confidence of the performance in a learning test (Dinsmore and Parkinson 2013).

## The present experiment

The current experiment aimed to investigate the effect of signaling in dependence on the extraneous cognitive load which was induced through the text font used in the learning environment. To do this, instructional texts were manipulated in terms of signaling (color cues vs. no color cues) and induced extraneous load (fluent text font vs. disfluent text font). According to the reviewed literature, signaling should lead to improved learning outcomes (van Gog 2014). Color cues can be viewed as attention-guiding features, which help learners to select important information, thus fostering generative processing (Mayer 2014).

### H1

Learners who receive an instructional text with color cues achieve higher learning scores than learners who receive an instructional text without color cues.

As well as the validation of the main effect of signaling (Schneider et al. 2018), the induced extraneous load’s moderating effect is especially interesting and relevant. With respect to the element interactivity effect, signaling is supposed to be especially beneficial in the condition with an illegible font and thus a high element interactivity through extraneous elements (Sweller 2010). Therefore, signaling might compensate for the negative effects of a high extraneous load. In contrast, if the extraneous load is low, no additional instructional support is needed to process the relevant information successfully. Thus, signaling has no influence on learning. On the contrary, extraneous load induction might trigger a more analytic and careful processing (e.g., Alter et al. 2007, Song and Schwarz 2008). Even if the extraneous load induction was especially strong, metacognitive benefits from the disfluency effect cannot be ruled out. Thus, two hypotheses were formulated:

### H2a [based on the concept of element interactivity]

Learners who receive an instructional text with an illegible font achieve higher learning scores when receiving additional color cues than learners who receive an instructional text with an illegible font and no color cues.

### H2b [based on the concept of desirable difficulty]

Learners who receive an instructional text with an illegible font achieve higher learning scores when receiving no additional color cues than learners who receive an instructional text with an illegible font and color cues.

Several process variables were investigated to obtain deeper insights into learning with instructional texts. Cognitive load was assessed to validate whether manipulating the illegible font succeeded in inducing a high extraneous load. In line with the disfluency effect, metacognitive variables were assessed to investigate whether the extraneous load manipulation had metacognitive benefits. Furthermore, subjective ratings for learning time and navigation behavior were measured to gain additional insights into the learning process, but since these variables were not in the main focus of the study, the procedures and results are displayed in “Appendix 2”).

## Methods

### Participants and design

Since recent studies and meta-analyses regarding signaling have reported at least medium effect sizes (e.g., Schneider et al. 2018), the estimated required sample size of this experiment is based on a medium effect. According to an a priori power analysis (*f* = .25; *α* = .05; 1 − *β* = 0.80; 2 × 2 design), at least 128 participants should be recruited for this study. Therefore, 143 university students from the Chemnitz University of Technology participated in the current experiment. Five participants had to be excluded due to technical and language problems. The remaining 138 students (85.5% female; age: *M* = 22.86; SD = 3.93) were included in the statistical analyses. The majority (71.7%) of the students had high school degrees and the remaining participants had academic degrees. Each participant received either 5€ or a 1-h course credit. No significant differences existed between the four treatment groups in terms of age (*p* = .19), gender (*p* = .70), educational level (*p* = .38) or prior knowledge (*p* = .55). The participants’ domain-specific prior knowledge was low to medium (mean percentage of correct answers in the prior knowledge test: *M* = 0.31, SD = 0.13). Each student was randomly assigned to one cell of a two-by-two factorial between-subjects design, by drawing lots (*signaling*: color cues versus no color cues and *Extraneous load induction*: illegible font versus legible font). Thirty-three students participated in the legible font and non-signaled condition, 34 students participated in the legible font and signaled condition, 35 students participated in the illegible font non-signaled condition and 36 students were assigned in the illegible font and signaled condition.

### Materials

The learning material consisted of an instructional text the content of which dealt with the emergence and characteristics of tsunamis, and protection against tsunamis. The text had 1340 words and was divided into six segments, which were presented on different Web sites. On average, 223.33 words were presented per segment. The participants could click on the forward or backward buttons in order to navigate through the Web sites. They could navigate and re-read the segments as often as they wanted and there was a finish button on the last page. Once this button had been clicked, the Web sites could no longer be accessed. An additional graphic was used on the last page to illustrate the emergence of tsunamis. The participants decided how long they wanted to learn themselves, but were told that they would be automatically redirected to the experiment’s next steps after a maximum of 20 min.

#### Signaling

Color cues were used to implement signaling in the texts. This operationalization was chosen because recent meta-analyses indicated that signaling could be successfully implemented in texts and that visual cues, like colors, were effective for encouraging learning (Richter et al. 2016; Schneider et al. 2018). The main concepts in the texts were marked in red (Hex Color Code: #ff6347), but the color of the text itself was not changed. The color was based on the color of classical text markers in order to strengthen external validity. Furthermore, the color was fairly bright to prevent any additional disfluency from the bad readability of the words. In total, 315 words were signaled (52.20 words per segment). This equated to 23.51% of the instructional text (the most important hard facts and key concepts).

#### Extraneous load induction

**Fig. 1:**
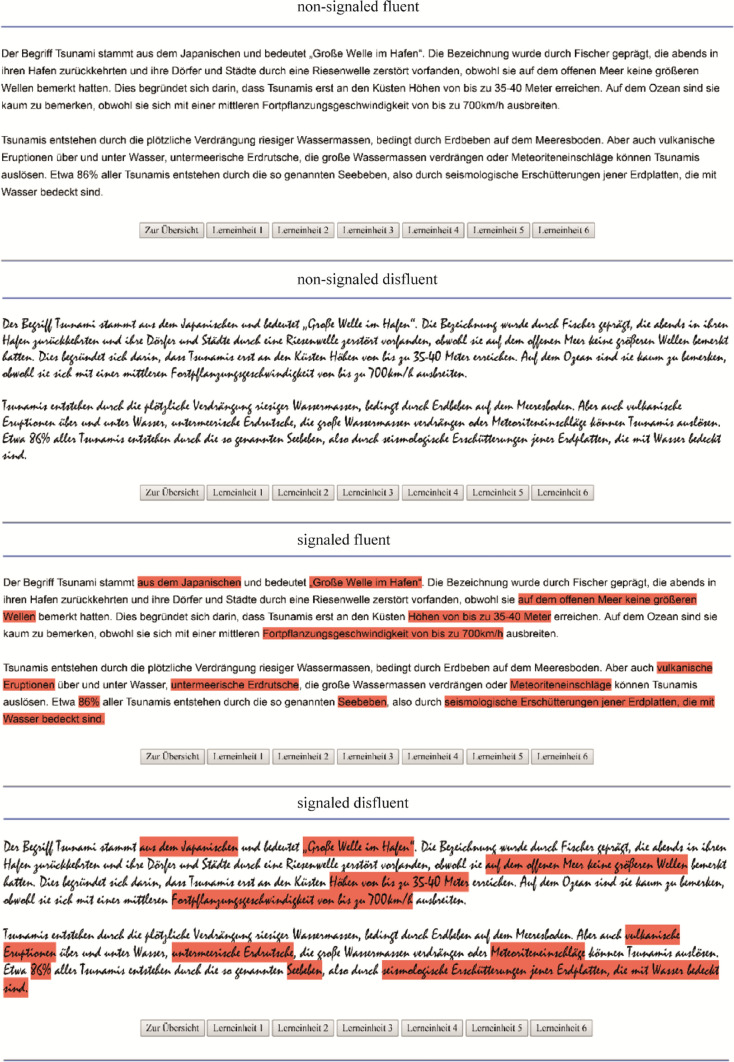
Screen example of the experimental manipulation

A pretest was conducted in order to determine how to induce a high extraneous load. Since extraneous loads can be achieved by using illegible fonts (e.g., Miele and Molden 2010; Rummer et al. 2016), different fonts were pretested by 49 participants in order to find a particularly hard-to-read font that induced an especially high disfluency. In the online test, the holo-alphabetic sentence (pangram): “Franz jagt im komplett verwahrlosten Taxi quer durch Bayern. (Franz hunts in a completely dilapidated taxi across Bavaria.)” was presented in 22 fonts; participants had to rate the items: *readability* (scale 1 = very bad; 7 = very good); *favor* (scale 1 = very bad; 7 = very good) and *suitability for learning* (scale 1 = not suitable; 7 = very suitable). Since each participant rated all the fonts, dependent measures analyses of variance (ANOVAs) were used for calculation. There was a significant effect for *readability* with a very large effect size, *F* (1, 21) = 71.56; *p* < .001; *η*_p_^2^ = .60. The “Arial” font had the best readability (*M* = 6.25; SD = 0.81) and “Mistral” the worst (*M* = 2.02; SD = 0.98). For *favor*, there was a significant effect with a very large effect size, *F* (1, 21) = 37.80; *p* < .001; *η*_p_^2^ = .45. Again, the “Arial” font had the highest score (*M* = 5.58; SD = 1.09) and “Mistral” the lowest (*M* = 2.46; SD = 1.57). There was a significant effect for *suitability* with a very large effect size, *F* (1, 21) = 144.63; *p* < .001; *η*_p_^2^ = .76). Again, the “Arial” font had the highest score (*M* = 5.80; SD = 0.73) and “Mistral” the lowest (*M* = 1.88; SD = 0.76). On the basis of these results, and since “Mistral” had already been used by Pieger and colleagues (2016), the Arial and Mistral fonts were used to implement good or bad text legibility in the current experiment. The “Times New Roman” font was used for the following questionnaires and learning tests because it scored highly for the three pretested characteristics readability (*M* = 5.58; SD = 1.37), evaluation (*M* = 4.67; SD = 1.28) and suitability (*M* = 4.88; SD = 1.05). This approach was chosen so that the font used for the test did not work as a memory cue. The instructional text is illustrated in Fig. 1.

### Measures

Revelle’s coefficient *ω* (e.g., McNeish 2018; Revelle and Zinbarg 2009) was chosen to calculate reliability estimates for all measures. The interpretation of the level of reliability is identical to that of Cronbach’s *α*. In the current manuscript, the focus is on the learning outcomes and the main explanatory variables (cognitive load and metacognition). Further assessed variables (navigation, learning and testing time, frustration and effort) are outlined in “Appendix 2.”

#### Cognitive load

Klepsch, Schmitz and Seufert’s cognitive load questionnaire (2017) was chosen because it refers to complexity of the content and the recognition of important information. Two items measured the intrinsic load (e.g., “This task was very complex.”) and three items measured the extraneous load (*ω* = .78; e.g., “It was exhausting to find the important information in this task.”). The participants had to rate the items on a 7-point Likert scale, ranging from 1 (absolutely wrong) to 7 (absolutely correct).

#### Metacognition and metacognitive accuracy

The procedure for assessing metacognitive judgments and metacognitive accuracy is based on Pieger and colleagues (2016). Ease of learning (EOL) was measured by the question, “How easy or difficult will it be to learn the text?” on a scale from 1 (very easy) to 101 (very difficult). Judgments of learning (JOL) were measured using the question, “What percentage of the questions about the text will you answer correctly?” on a scale from 1 (very easy) to 101 (very difficult). The JOL question was implemented for each text segment. Retrospective confidence (RC) was measured by the question, “How confident are you that your answer is correct?” on a scale from 1 (unconfident) to 101 (confident). The RC questions were implemented after every retention and transfer question.

Metacognitive accuracy was calculated as absolute accuracy and relative accuracy. The three metacognition scores (EOL, JOL and RC ratings) and the overall learning score were first z-standardized. For the absolute accuracy calculation, the performance score was subtracted from the three metacognition scores. For the relative accuracy calculation, the within-person Goodman–Kruskal gamma correlations between the metacognitive judgments and the retention and transfer test item scores were calculated.

#### Learning and prior knowledge

Prior knowledge was measured by one item, “Please describe how tsunamis emerge.” using an open-answer format. The item was evaluated by two independent raters. The inter-rater reliability (i.e., the intra-class-correlation coefficient (ICC) of aggregated prior knowledge score) was high, ICC (2, *k*) = 995; *F*(137, 137) = 189.33; *p* < .001. A maximum of four points could be achieved. Three further items (*ω* = .77; “Tsunamis were taught in school.”; “I have dealt with the topic of tsunamis in my spare time.”; “I have dealt with the emergence of tsunamis before.”) were implemented and rated on a scale ranging from 1 (not at all) to 7 (very detailed), in order to assess the participants’ prior experience of the topic.

Learning was measured using two scales: retention and transfer. According to Mayer (2014), retention can be defined as “remembering” content which has been explicitly presented in an instructional text. Transfer knowledge is defined as “understanding.” The learners had to solve novel problems which were not explicitly presented in the instructional text by using the acquired knowledge (Mayer 2014). Retention (*ω* = .65) was measured with a 10-item multiple-choice questionnaire. The students were asked to choose from six possible answers; each question could have one to six correct answers. They received points for selecting the correct answers and for not selecting the wrong ones and were therefore able to score up to six points per question. The questions referred to information that had been explicitly presented in the text, such as “Which statements about the causes of tsunamis are correct?” An additional open question (“Name three requirements for tsunamis.”) was also implemented to measure retention. The inter-rater reliability was perfect. The same question format was used to obtain the transfer scores. A 5-item scale was created in which every item presented a new scenario and the items had three to seven possible answers. Two open question were also used (e.g., “Calculate the speed of a tsunami with the formula you learned in the text.”). The inter rater reliability was perfect. Every item dealt with a different subtopic from the learning material, in order to assess transfer performance to greatest possible extent. In consequence, the reliability of the scale cannot be reported because the items assessed nearly independent subdomains of the overall knowledge; a detailed applicable knowledge in one domain does not necessarily mean that the participants could apply their knowledge correctly to other items.

### Procedure

A computer laboratory at the university with ten identical computers was prepared before each experimental session. 24-inch monitors were provided for this setup, and the online questionnaire was opened at each workstation. The online learning environments could be accessed on the SoSci Survey (Leiner 2016) by entering a particular link. Up to ten participants were tested simultaneously. Sight-blocking partition walls were used to ensure that the students worked independently. At the beginning of the experiment, the participants were told that the experiment was an instructional study on a science topic and were asked to answer a previous knowledge test. Then, they were given the link and asked to take a preliminary look at the learning environment and the learning text. After 2 s, the participants were automatically redirected to a questionnaire and had to evaluate the EOL item. The learning phase then began. The students had to learn the six segments of the online environment at a learner’s pace (with a maximum duration of 20 min). They were able to navigate freely between the individual learning segments. When they had finished the learning phase, the students had to rate the JOL items for each segment. The dependent variables were measured after finishing the learning phase. The cognitive load was assessed first, followed by the two NASA-TLX items. Afterward, as in the previous research, a filler (distraction) task was implemented (see Weissgerber and Reinhard 2017). Participants were instructed to work on different tasks, but not to put a special effort into solving the task. The filler task consisted of questions about the capitals of German states, media preferences, lifestyle and food, a hidden object game, a counting task and a math test. The filler task lasted 25 min and was implemented to ensure rather robust learning effects. There is a lot of research in terms of multimedia learning that only assesses and interprets rather short-term learning effects which might be explained by the fact that the knowledge is still present in the working memory. We wanted to know whether rather robust learning effects could be observed and thus wanted to investigate whether the knowledge is still present when the working memory was distracted. Afterward, retention and transfer were measured. An RC item had to be answered after every retention and transfer question. Finally, the students had to answer a demographic questionnaire. When all the tests had been completed, the participants could leave the room. The experiment lasted a total of 60 min.

## Results

Multivariate analyses of covariance (MANCOVAs) were conducted in order to investigate differences between the experimental groups. Signaling (signaled versus non-signaled) and extraneous load (ECL) induction (illegible font versus legible font) were used as independent variables for all the analyses and the students’ prior knowledge and prior experience were used as covariates. The test assumptions were examined, and only significant violations of these assumptions were reported. The effect sizes for all differences were only reported if they attained significance (*p* < .05). First, the learning outcomes were investigated in order to support or reject the hypotheses and to answer the research question. Next, the process variables were studied in order to obtain deeper insights into the learning process. Again, the focus is on the learning outcomes and the most important explanatory variables (cognitive load, metacognition). Results regarding navigation, learning and testing time, frustration and effort are displayed in “Appendix 2.” The descriptive results for all dependent variables are shown in Table 1, and bar diagrams for all dependent measures are displayed in “Appendix 1.”

**Table 1:** Mean and standard deviations of all dependent variables for the four experimental groups

	Experimental groups							
	Legible font				Illegible font			
	Signaled (*N* = 34)		Non-signaled (*N* = 33)		Signaled (*N* = 36)		Non-signaled (*N* = 35)	
	*M*	SE	*M*	SE	*M*	SE	*M*	SE
ICL	8.80	0.50	8.26	0.51	10.03	0.48	9.60	0.49
ECL	8.54	0.63	12.39	0.64	14.30	0.61	16.31	0.63
Retention	38.74	0.95	37.89	0.96	36.55	0.92	37.19	0.94
Transfer	12.74	0.55	11.46	0.56	11.94	0.53	10.51	0.54
EOL	66.69	3.88	59.93	3.94	73.06	3.76	87.82	3.85
JOL	59.67	2.93	54.88	2.97	44.80	2.84	45.90	2.90
RC	49.46	3.30	41.09	3.35	38.16	3.20	41.57	3.27
*Absolute accuracy*								
EOL	− 0.52	0.24	− 0.52	0.24	0.14	0.23	0.85	0.24
JOL	0.17	0.20	0.18	0.20	− 0.27	0.19	− 0.10	0.20
RC	0.05	0.19	− 0.09	0.19	− 0.12	0.18	0.15	0.19
*Relative accuracy*								
EOL (gamma)	− 0.16	0.11	− 0.17	0.11	0.03	0.12	0.18	0.15
JOL (gamma)	0.16	0.12	0.08	0.12	0.38*	0.08	0.25*	0.10
RC (gamma)	0.17	0.10	0.16	0.12	0.55*	0.10	0.31*	0.10

ICL scores ranged from 2 to 14. ECL scores ranged from 3 to 21. The retention score ranged from 0 to 63. The transfer score ranged from 0 to 23. EOL, JOL and RC Scores ranged from 1 to 101. Scores are controlled for prior knowledge and prior experience

*ICL* Intrinsic cognitive load, *ECL* extraneous cognitive load, *EOL* ease of learning, *JOL* judgment of learning, *RC* retrospective confidence, *M* = mean scores, *SD* standard deviation

*Gamma coefficient reached significance, *p* < .05

### Learning outcomes

A MANCOVA was conducted using retention and transfer score as dependent measures. Prior knowledge was a significant covariate, Wilks’s Λ = 0.92; *F*(2, 131) = 5.79; *p* = .004; *η*_p_^2^ = .08, but prior experience was not: Wilks’s Λ = 0.999; *F*(2, 131) = 0.08; *p* = .93. A significant effect with a small effect size was found for signaling: Wilks’s Λ = 0.95; *F*(2, 131) = 3.49; *p* = .03; *η*_p_^2^ = .05. However, no significant effect was found for font legibility, Wilks’s Λ = 0.97; *F*(2, 131) = 1.72; *p* = .18, and no interaction was apparent: Wilks’s Λ = 0.99; *F*(2, 131) = 0.43; *p* = .66.

Follow-up ANCOVAs were conducted in order to obtain a detailed insight into the signaling effect. No significant effect was found for retention: *F*(1, 132) = 0.01; *p* = .91. A significant main effect with a small to medium effect size could be found for transfer: *F*(1, 132) = 6.24; *p* = .01; *η*_p_^2^ = .05. Transfer performance was enhanced in the signaled conditions, in contrast to the non-signaled conditions.

### Cognitive load

In order to obtain detailed insights into cognitive processes during learning, a MANCOVA was conducted using intrinsic load and ECL as dependent measures. Prior knowledge was a significant covariate, Wilks’s Λ = 0.92; *F*(2, 131) = 5.93; *p* = .003; *η*_p_^2^ = .08, but prior experience was not: Wilks’s Λ = 0.98; *F*(2, 131) = 1.69; *p* = .19. A significant effect with a large effect size was found for signaling, Wilks’s Λ = 0.84; *F*(2, 131) = 12.80; *p* < .001; *η*_p_^2^ = .16. A significant effect with a large effect size was found for font legibility, Wilks’s Λ = 0.69; *F*(2, 131) = 28.96; *p* < .001; *η*_p_^2^ = .31. No interaction could be observed: Wilks’s Λ = 0.98; *F*(3, 131) = 1.17; *p* = .32.

Follow-up ANCOVAs were conducted in order to obtain detailed insights into both cognitive load facets. In terms of intrinsic load, no effect was found for signaling, *F*(1, 132) = 0.96; *p* = .33; however, a significant effect with a small effect size was found for font legibility, *F*(1, 132) = 6.54; *p* = .01; *η*_p_^2^ = .05, and the intrinsic load was enhanced in the illegible font conditions, compared to the legible font conditions. No interaction could be observed, *F*(1, 132) = 0.01; *p* = .91. In terms of ECL, a significant effect with a large effect size was found for signaling, *F*(1, 132) = 21.91; *p* < .001; *η*_p_^2^ = .14. ECL was reduced in the signaling conditions compared to the non-signaled conditions. A significant main effect with a large effect size was found for font legibility, *F*(1, 132) = 57.28; *p* < .001; *η*_p_^2^ = .30. ECL was enhanced in the illegible font conditions, compared to the legible font conditions. Therefore, the induction of ECL through an illegible font can be viewed as successful. No interactions could be observed, *F*(1, 132) = 2.17; *p* = .14)

### Metacognition and metacognitive accuracy

In order to investigate the metacognitive measures, a MANCOVA was conducted using EOL, mean JOL and mean RC score as dependent measures. Prior knowledge was a significant covariate, Wilks’s Λ = 0.92; *F*(2, 130) = 3.74; *p* = .01; *η*_p_^2^ = .08, but prior experience was not: Wilks’s Λ = 0.99; *F*(2, 130) = 0.31; *p* = .82. There was no significant effect for signaling, Wilks’s Λ = 0.99; *F*(2, 130) = 0.54; *p* = .66. A significant main effect with a large effect size was found for font legibility, Wilks’s Λ = 0.78; *F*(2, 130) = 12.13; *p* < .001; *η*_p_^2^ = .22, and an interaction with a medium effect size could be observed, Wilks’s Λ = 0.92; *F*(2, 130) = 4.04; *p* = .01; *η*_p_^2^ = .09.

Follow-up ANCOVAs were conducted in order to investigate the main effect of font legibility and the interaction further. In terms of EOL, a significant main effect with a medium effect size was found for font legibility, *F*(1, 132) = 19.09; *p* < .001; *η*_p_^2^ = .13. EOL was enhanced in the illegible font conditions, compared to the legible font conditions. A significant interaction with a medium effect size could be found: *F*(1, 132) = 7.86; *p* = .01; *η*_p_^2^ = .06. In the non-signaled conditions, an illegible font led to higher EOL scores that a legible font. In the signaled conditions, the values only differed slightly (see “Appendix 1”). In terms of JOL, a significant main effect with a medium effect size was found font legibility: *F*(1, 132) = 16.26; *p* < .001; *η*_p_^2^ = .11. JOL was reduced in the illegible font conditions, compared to the legible font conditions. No interaction could be observed, *F*(1, 132) = 1.04; *p* = .31. In terms of RC, neither a significant main effect for font legibility, *F*(1, 132) = 2.63; *p* = .11, nor an interaction could be found: *F*(1, 132) = 3.26; *p* = .07.

In order to investigate metacognitive accuracy, a MANCOVA was conducted using the EOL accuracy, JOL accuracy and RC accuracy scores as dependent measures. Neither prior knowledge, Wilks’s Λ = 0.95; *F*(2, 130) = 2.52; *p* = .06, nor prior experience, Wilks’s Λ = 0.99; *F*(2, 130) = 0.30; *p* = .82, was a significant covariate. There was no main effect for signaling: Wilks’s Λ = 0.98; *F*(2, 130) = 0.77; *p* = .51. A significant main effect with a large effect size could be found for font legibility: Wilks’s Λ = 0.78; *F*(2, 130) = 12.20; *p* < .01; *η*_p_^2^ = .22. No interaction could be observed: Wilks’s Λ = 0.98; *F*(2, 130) = 1.10; *p* = .35.

Follow-up ANCOVAs were conducted to obtain deeper insights into the significant effect found for font legibility. In terms of EOL accuracy a significant main effect with a medium effect size could be found, *F*(1, 132) = 17.38; *p* < .001; *η*_p_^2^ = .12. Students working with the illegible font had higher accuracy scores compared to those with the legible font. *t*-tests against zero (no over- or under-confidence) revealed that no condition was highly accurate. Students in the illegible font conditions were significantly under-confident, *t*(70) = 2.73, *p* = .01, whereas participants in the legible font conditions were significantly overconfident, *t*(66) = − 2.37, *p* = .02. There was no significant effect in terms of JOL accuracy, *F*(1, 132) = 3.23; *p* = .08, and RC accuracy, *F*(1, 132) = 0.04; *p* = .85. *T* test against zero revealed nonsignificant results.

In order to investigate relative accuracy, the gamma coefficients were calculated (see Table 1). Under the legible font conditions, no significant correlations could be found between the metacognitive judgments and the overall learning scores. In contrast, there were significant positive correlations between metacognitive judgments (JOL and RC) and the overall learning scores under the illegible font conditions. This indicates that students in the legible font conditions could not evaluate their learning and test performance accurately, whereas students in the illegible font conditions were able to estimate their performance more precisely, both during learning and the following retention and transfer test.

## Discussion

The goal of the current study was to investigate how signaling affects learning and learning-relevant processes, in interdependence with an extraneous cognitive load which was induced by using illegible text fonts. The results revealed that signaling significantly enhanced transfer, but not retention performance. Therefore, hypothesis 1 was partially supported. Extraneous load induction had no significant effect on learning scores, even when the retention and transfer scores were descriptively reduced in the high extraneous load condition group. Concerning hypotheses 2a and 2b, no interaction, and therefore no moderation, occurred regarding both learning scores. In consequence, hypotheses 2a and 2b must be rejected.

The main effect of signaling on transfer can be explained by considering the CLT; color cues worked as attention-guiding features within the instructional text. The learners were assisted in identifying and selecting relevant. The attention of the learners was directed to the most important information which was necessary to build a complex schema. Additionally, learners were not distracted by less relevant information or overloaded by too much information at once. This is reflected in the extraneous load scores, which were lowered in the signaling condition group. The results suggest that signaling can be explained by the inherent cognitive benefits mentioned, since metacognitive judgments and accuracy were not influenced by color cues. Yet, two important restrictions have to be discussed. First, retention scores were not affected by color cues, and in consequence, the signaling effect could only be partially replicated. A possible explanation might be the low complexity of the learning material itself. Even if participants had low prior knowledge, the element interactivity of the material might be too low to overtax learners in terms of simple recognition of information. Color cues could not enhance retention performance since learners had only marginal difficulties in remembering basic facts, but were effective in helping the learner to obtain a comprehensive mental model which was necessary to adapt their knowledge to other problems. Thus, information got processed more deeply and constructed higher-quality schemata in their long-term memory. Second, according to the results of the additional measures (see “Appendix 2”), signaling was only beneficial for learners who had at least some prior knowledge. This supports the results of a recent meta-analysis by Schneider and colleagues (2018), who pointed out that learners with prior knowledge are better able to cope with the additional information provided by signaling, than those with low prior knowledge.

An illegible text font increased extraneous load with a high effect size. Therefore, the induction can be viewed as successful. Interestingly, intrinsic load was enhanced through an illegible font as well. The items from the used ICL scale (Klepsch et al. 2017) deal with the perceived complexity of the material. When learners had trouble, reading the material they might also think that the material is overly complex which might have led to the increased ICL rating. The induction of an extraneous load does not seem to influence learning outcomes in general. This is especially interesting since the perceived cognitive load was increased in the high extraneous load conditions. Seufert and colleagues’ (2017) findings in terms of the learning–inhibiting effect of a high disfluency could not be replicated.

This contradictory finding might be explained by considering metacognitive variables. On the one side, the enhanced ECL in learning environments had potential negative effects on learning. In line with recent research (e.g., Pieger et al. 2016), inducing extraneous load by using an illegible text font reduced confidence at the time the students first saw the learning environment. Learners receiving an illegible font were significantly under-confident and not accurate in regard to their later learning outcomes. Furthermore, learners reported an enhanced frustration, working with the illegible material (see “Appendix 2”). This might provide further support for the results reported by Reber, Schwarz and Winkielman (2004), who pointed out that disfluent perceptions of objects led to negative responses toward them.

On the other side, the overall EOL score was particularly enhanced by the extraneous load manipulation, which, in turn, encouraged metacognitive monitoring. Even if the EOL accuracy was reduced, the high extraneous load in the form of an illegible font acted as a trigger for more analytic and deliberate monitoring during learning (Alter et al. 2007), which was reflected in the relative metacognitive accuracy regarding JOL and RC. In contrast to the students with the low-extraneous-load conditions, those in the high extraneous load conditions were aware of potential difficulties regarding the learning task and thus were able to estimate their performance correctly, both during learning and the following retention and transfer test. In consequence, the metacognitive benefits of a high extraneous load might compensate for the negative cognitive effects of such a load so that the learning outcomes were not influenced. This is further supported by considering additional variables (see “Appendix 2”). Even if frustration is enhanced through an illegible text font, the learners reported that they put more effort into understanding the learning content.

The results of this experiment support the assumption that the induction of extraneous load is not detrimental to learning in general. Instead, the higher extraneous load induced through the manipulation of perceptual disfluency led to more elaborate metacognitive monitoring concerning JOL and RC which at least compensate for the negative effects of a high ECL. These metacognitive benefits have an effect even when the disfluency manipulation is particularly strong (Seufert et al. 2017).

No interaction effects regarding learning could be found. Statistically, signaling seems to be beneficial for transfer, independent of the induced extraneous load. However, interaction effects occurred in terms of EOL judgments. Especially in the high-extraneous-load condition group, signaling reduced EOL judgments, indicating that signaling might work as a visual cue for reducing perceptual disfluency. Learning behavior changed as a result (see “Appendix 2”). Students working under the high-extraneous-load condition with implemented color cues navigated through the pages more often without taking any more time for the learning itself than those working under the high-extraneous-load condition without color cues. These results patterns reversed in the low-extraneous-load conditions group. Implementing signals in learning environments with a high extraneous load might weaken the learning-relevant metacognitive monitoring processes and therefore change learning behavior. This is especially interesting since descriptively, the retention scores were enhanced in the low extraneous load—signaled—and high extraneous load—non-signaled conditions—indicating that extraneous load might have an influence on the effects of signaling. Implementing signals in disfluent learning environments might weaken the learning-relevant metacognitive monitoring processes and therefore change learning behavior. Nevertheless, the results regarding interactions in the learning scores did not reach significance, and therefore, these interpretations have to be viewed with caution.

### Implications

On the theoretical side, the signaling principle is supported partially. Implementing attention-guiding cues in an instructional text can foster learning transfer, but not retention processes. Inducing an extraneous load through the learning environment does not seem to moderate the effect of signaling on learning outcomes. This is a theoretical implication which is in contrast to the concept of element interactivity through extraneous elements. Nevertheless, the fact that learners should have at least a low or moderate level of prior knowledge in order to cope with the additional load from the implemented signals must be taken into account. The element interactivity effect could not be replicated by manipulating element interactivity by inducing an extraneous load, instead of manipulating an intrinsic load. Thus, extending the concept of element interactivity to an extraneous load (Sweller 2010) might be subject to additional boundary conditions. For example, the learners’ prior knowledge is important. If they have no prior knowledge, inducing load through illegible fonts impairs their learning compared to those learners who have a low to medium degree of prior knowledge. Furthermore, the induction of extraneous load by manipulating visual presentation can lead to metacognitive benefits. These benefits can compensate for the negative effects of a multimedia learning environment’s learning–inhibiting design.

On the practical side, designers should be encouraged to signal important parts of instructional texts, to foster learning transfer and to reduce the learners’ irrelevant cognitive load. It is irrelevant whether instructors aim to create a simple learning environment or one that is more complex with potential extraneous cues. Signaling can be implemented in order to enhance transfer and reduce the subjective irrelevant load. The font is not significant for learning success, but designers should be aware that illegible fonts lead to frustration and a higher perceived load. If the learning material induces an extraneous load, designers should be particularly aware that signaling changes the learning behavior, compared to a text without it.

### Limitations

The manipulation might have been too weak in terms of extraneous load. Although the fonts used were pretested, a stronger extraneous load manipulation might lead to differences in the effects of signaling, since the metacognitive benefits would be unable to compensate for the induced load. According to Seufert and colleagues (2017), multiple methods of inducing disfluency should be considered, because perceptual fluency, at the level used in this study, might not be a diagnostic cue for performance (e.g., Dunlosky and Thiede 2013). In consequence, it is difficult to generalize the current results to other kinds of extraneous load induction. To generalize the finding from the current investigation, it would be necessary to implement and investigate various manipulations. In addition, the disfluent font, which was used in this experiment, looks more like a handwritten font than the fluent font. The perception of it as a handwritten font could trigger social processes since the learner might feel that they were being addressed by an actual human being with their own intentions. This might trigger “para” social communication processes and foster processes, like attention (e.g., Grice 1975), thus further compensating for the negative effect of a high extraneous load. Recent meta-analyses (e.g., Schneider et al. 2018) have indicated that different types of signaling (e.g., color coding, organizational signals) have different influences on learning. Therefore, it is difficult to generalize the results for other signaling modes. Another limitation must be mentioned regarding the exploratory analyzes in terms of prior knowledge; dividing the participants into two groups and investigating them separately led to a significant drop in statistical power. This must be taken into account, especially when discussing the descriptive trends of individual learning measures. Finally, the filler task has to be discussed. Even if the filler task was just implemented to distract the participants from the material, it is possible that some participants put special effort into it. Thus, it is possible that retroactive interference occurred (e.g., Craig et al. 2015). Participants who put more effort into the task could have been adversely affected, and thus, learning performance could be lower.

### Future directions

Based on the limitations of the current study, future research should focus on different types of signaling modes, signaling types and the induction of extraneous load. Extraneous load was induced through the text font in this study, but other methods, such as the use of dialects (Sweller 2010) or decorative pictures (Rey 2012), should also be taken into account. Different types of learning material should be investigated in this context. Signaling implemented in simple or potentially irritating graphics might be a promising field for future research. Finally, the current study was carried out with a student sample. In order to strengthen external validity, additional studies should be carried out with primary or secondary students, since these age-groups often work primarily with instructional textbooks.

## Appendix 1: Bar diagrams for dependent variables (*M* and SE are displayed)

Effect of signaling and text font on ICL.

Effect of signaling and text font on ECL.

Effect of signaling and text font on retention.

Effect of signaling and text font on transfer.

Effect of signaling and text font on EOL.

Effect of signaling and text font on JOL.

Effect of signaling and text font on RC.

Effect of signaling and text font on EOL accuracy (absolute).

Effect of signaling and text font on JOL accuracy (absolute).

Effect of signaling and text font on RC accuracy (absolute).

## Appendix 2: Additional measures and results

### Additional measures

#### Frustration and effort

Two items from the NASA-TLX (Hart and Staveland 1988) were taken to assess the subjective perception of learning. One item (“How frustrated did you feel during the task?”) measured perceived frustration, and a second item (“How hard did you have to work to accomplish the task?”) measured invested effort. The items were rated on sliders, from 1 (low frustration/effort) to 20 (high frustration/effort).

#### Time and navigation

In order to gain insights, the objective learning characteristics such as learning time, testing time and navigation were assessed. The learning and testing times were tracked by the online system. The output was in seconds. For navigation, the online system tracked how often the students clicked the forward or backward buttons. Therefore, the navigation variable reflects how often the participants switched between the six learning segments.

### Results regarding the additional measures

#### Frustration and effort

A MANCOVA was conducted using frustration and effort as dependent measures. Neither prior knowledge, Wilks’s Λ = 0.97; *F*(2, 131) = 2.07; *p* = .13, nor prior experience, Wilks’s Λ = 0.98; *F*(2, 131) = 1.41; *p* = .25 were significant covariates. No effect was found for signaling, Wilks’s Λ = 0.99; *F*(2, 131) = 0.40; *p* = .67. A main effect with a large effect size was found for font legibility: Wilks’s Λ = 0.80; *F*(2, 131) = 16.47; *p* < .001; *η*_p_^2^ = .20. No interaction could be observed: Wilks’s Λ = 0.998; *F*(2, 131) = 0.10; *p* = .90.

Follow-up ANCOVAs were conducted in order to determine the significant effect of font legibility. A significant main effect with a large effect size was found for frustration, *F*(1, 132) = 30.44; *p* < .001; *η*_p_^2^ = .19. Frustration was enhanced in the illegible font conditions, compared to the legible font conditions. A significant effect with a medium effect size was found for effort, *F*(1, 132) = 16.28; *p* < .001; *η*_p_^2^ = .11. Effort was enhanced in the illegible font conditions, compared to the legible font conditions.

#### Time and navigation

A MANCOVA was conducted using learning time, testing time and navigation as dependent measures. Prior knowledge was a significant covariate, Wilks’s Λ = 0.94; *F*(2, 130) = 2.93; *p* = .04; *η*_p_^2^ = .06, but not prior experience, Wilks’s Λ = 0.98; *F*(2, 130) = 0.71; *p* = .55. No significant main effect was found for signaling, Wilks’s Λ = 0.99; *F*(2, 130) = 0.25; *p* = .86. A significant main effect with a large effect size was found for font legibility, Wilks’s Λ = 0.81; *F*(2, 130) = 9.98; *p* < .001; *η*_p_^2^ = .19, and a significant interaction with a medium effect size could be observed, Wilks’s Λ = 0.93; *F*(2, 130) = 3.30; *p* = .02; *η*_p_^2^ = .07.

Follow-up ANCOVAs were conducted in order to gain detailed insights into the effect of font legibility and the interaction. No effect was found for font legibility in terms of learning time, *F*(1, 132) = 0.003; *p* = .96, and no interaction could be observed, *F*(1, 132) = 2.44; *p* = .12. In terms of testing time, a significant main effect with a medium effect size was found for font legibility, *F*(1, 132) = 8.67; *p* = .004; *η*_p_^2^ = .06. Students working under the illegible font conditions completed the learning test faster than those with the legible font conditions. Furthermore, a significant interaction with a small effect size could be found, *F*(1, 132) = 7.27; *p* = .01; *η*_p_^2^ = .05. For the conditions with signaling, testing time only differs slightly regarding font legibility. In the conditions without signaling, the students working with an illegible font completed the learning test faster than those with the legible font. In terms of navigation, a significant effect with a medium effect size was found for font legibility, *F*(1, 132) = 13.97; *p* < .001; *η*_p_^2^ = .10. Students working with an illegible font navigated less compared to those with the legible font. A descriptive indication for an interaction could be found, *F*(1, 132) = 3.85; *p* = .052. Descriptively, the main effect of font legibility was stronger in the non-signaled conditions than in the signaled conditions.

### Exploratory analyses regarding prior knowledge

All the previous analyses controlled for prior knowledge, but did not explicitly analyze it. Nevertheless, the participants had low to medium prior knowledge. Domain-specific knowledge could interact with the manipulated factors, since it could reduce element interactivity (Chen et al. 2017). Thus, signaling might be less effective (perhaps even detrimental) for participants with higher levels of prior knowledge (Kalyuga 2007) or especially beneficial for high-prior-knowledge learners (Schneider et al. 2018). Furthermore, learners with no prior knowledge might have difficulties with a high induced ECL. First, the participants were divided into two groups. The first group consisted of participants with no prior knowledge (*N* = 58) and the other group, of those with at least some prior knowledge (*N* = 80).

Investigating only learners with no prior knowledge, a MANCOVA with retention and transfer as dependent variables and prior experience as the covariate, revealed that prior experience was no significant covariate: Wilks’s Λ = 0.98; *F*(2, 52) = 0.58; *p* = .57. No significant effect was found for signaling: Wilks’s Λ = 0.95; *F*(2, 52) = 1.37; *p* = .26. A significant effect with a medium effect size was found for font legibility, Wilks’s Λ = 0.89; *F*(2, 52) = 3.18; *p* = .05, *η*_p_^2^ = .11 and no interaction could be observed: Wilks’s Λ = 0.98; *F*(2, 52) = 0.56; *p* = .58. Follow-up ANCOVAs were conducted in order to gain a detailed insight into the effect of font legibility. No significant effect was found for retention, *F*(1, 53) = 2.35; *p* = .13, but descriptively, the retention scores were lower under the illegible font conditions. An illegible font significantly reduced transfer performance with a medium effect size, *F*(1, 53) = 5.12; *p* = .03; *η*_p_^2^ = .09.

When investigating the participants with at least some prior knowledge, a MANCOVA with retention and transfer as dependent variables and prior experience as the covariate, revealed that prior experience was no significant covariate: Wilks’s Λ = 0.99; *F*(2, 74) = 0.45; *p* = .64. A descriptive trend was found for signaling: Wilks’s Λ = 0.93; *F*(2, 74) = 2.92; *p* = .06. No effect was found for font legibility, Wilks’s Λ = 0.997; *F*(2, 74) = 0.11; *p* = .90, and no interaction could be observed: Wilks’s Λ = 0.97; *F*(2, 74) = 1.02; *p* = .36. Follow-up ANCOVAs were conducted in order to gain a detailed insight into the descriptive trend regarding signaling. No significant effect was found for retention, *F*(1, 75) = 2.40; *p* = .13, but descriptively, the retention scores were enhanced in the signaling condition. Signaling significantly enhanced transfer performance, with a low to medium effect size: *F*(1, 75) = 5.82; *p* = .02; *η*_p_^2^ = .07.

**Table:** Mean and standard deviations of all additional dependent variables for the four experimental groups

	Experimental groups							
	Legible font				Illegible font			
	Signaled (*N* = 34)		Non-signaled (*N* = 33)		Signaled (*N* = 36)		Non-signaled (*N* = 35)	
	*M*	SE	*M*	SE	*M*	SE	*M*	SE
Frustration	12.02	0.57	15.55	0.57	14.54	0.55	14.82	0.56
Effort	7.44	0.82	7.86	0.83	11.71	0.79	12.72	0.81
Learning time	874.57	40.51	908.80	41.07	939.40	39.20	848.57	40.16
Testing time	1069.09	44.16	1171.90	44.76	1055.44	42.73	922.99	43.77
Navigation	15.13	0.90	16.21	0.91	13.48	0.87	11.07	0.89

Frustration and effort scores ranged from 0 to 20. Scores are controlled for prior knowledge and prior experience

Effect of signaling and text font on frustration.

Effect of signaling and text font on effort.

Effect of signaling and text font on learning time in seconds.

Effect of signaling and text font on testing time in seconds.

Effect of signaling and text font on navigation.
