# Correction to: The effect of signaling in dependence on the extraneous cognitive load in learning environments

**DOI:** 10.1007/s10339-021-01051-4

**Published:** 2021-08-10

**Authors:** Maik Beege, Steve Nebel, Sascha Schneider, Günter Daniel Rey

**Affiliations:** Psychology of Learning with Digital Media, Faculty of Humanities, Chemnitz University of Technology, Straße der Nationen 12, 09111 Chemnitz, Germany

## Correction to: Cognitive Processing (2021) 22:209–225 https://doi.org/10.1007/s10339-020-01002-5

The article “The effect of signaling in dependence on the extraneous cognitive load in learning environments”, written by Maik Beege, Steve Nebel, Sascha Schneider and Günter Daniel Rey, was originally published Online First without Open Access. After publication in volume 22, issue 2, page 209–225 the author decided to opt for Open Choice and to make the article an Open Access publication. Therefore, the copyright of the article has been changed to © The Author(s) 2020, and the article is forthwith distributed under the terms of the Creative Commons Attribution 4.0 International License, which permits use, sharing, adaptation, distribution and reproduction in any medium or format, as long as you give appropriate credit to the original author(s) and the source, provide a link to the Creative Commons licence, and indicate if changes were made. The images or other third party material in this article is included in the article’s Creative Commons licence, unless indicated otherwise in a credit line to the material. If material is not included in the article’s Creative Commons licence and your intended use is not permitted by statutory regulation or exceeds the permitted use, you will need to obtain permission directly from the copyright holder. To view a copy of this licence, visit http://creativecommons.org/licenses/by/4.0.

Open access funding enabled and organized by Projekt DEAL.

Original article has been corrected.

